# Anatomy and Physiology

**Published:** 1837-10

**Authors:** 


### PART FOURTH.

### Selections from the British Journals.

(FOR THE QUARTER ENDING SEPTEMBER 30, 1837.)

---

### ANATOMY AND PHYSIOLOGY.

*On the first changes in the Ova of the Mammifera, in consequence of Impregnation; and of the mode of Origin of the Chorion.* By THOMAS WHARTON JONES, Esq. (*Read before the Royal Society, April* 27, 1837.)

THE author having, in a former paper, described the structure of the unimpregnated ovum of mammiferous animals, now proceeds to investigate the changes which the ovum undergoes in consequence of impregnation. In the rabbit, the first perceptible difference is the addition of a thick gelatinous matter surrounding the parts of which the ovum was composed in its original state, and apparently derived from the ovaries. In the progress of development the vitellary membrane gives way, as happens in the ova of the newt, and of many of the oviparous animals. The gelatinous envelope acquired in the ovary, and which is more especially circumscribed and defined after impregnation, constitutes the only covering of the vascular blastoderma, after the giving way of the vitellary membrane, and afterwards forms the chorion, which in rodent animals, at a further stage of development, presents itself under the form of a thin and transparent membrane, very similar to the vitellary membrane of a bird's egg, and situated immediately outside the non-vascular and reflected layer of the umbilical or erythoid vesicle. The author draws similar conclusions with regard to the development of the human ovum.

The second part of the paper relates to the changes taking place in the vitellus, the inferences concerning which are deduced chiefly from observations of the development of the ova of batrachian reptiles. The author concludes that the disappearance of the germinal vesicle is prior to impregnation. In the newt, the vesicle, at first imbedded in the substance of the yelk, gradually approaches the surface, until its situation is immediately underneath the vitellary membrane: its coat, having now become very soft, gives way, allowing the contained fluid to be effused on the surrounding surface of the yelk; and the small depression in which the vesicle was lodged now forms the cicatricula. The effused fluid gives a degree of consistence to the matter composing the surface of the yelk, and thus promotes the formation of the blastoderma. In the frog, the surface of the yelk becomes every day more and more broken up, and the resulting crystalline forms described by Prevost and Dumas become smaller and smaller, until the surface of the black blastoderma appears under a magnifying glass like shagreen. The blastoderma, consisting of an aggregation of clear globules, different from those of the rest of the yelk, is now fully formed, and has extended itself so as to close in the white spot. The change which takes place in the yelk of the bird's egg appears to be limited to the neighbourhood of the cicatricula.

*Proceedings of the Royal Society,* No. xxix.

---

*On the Temperature of Insects, and its Connexion with the Functions of Respiration and Circulation.* By GEORGE NEWPORT, Esq. (*Read before the Royal Society, June* 15, 1837.)

THE author states at the commencement of his paper, that, although it has been long known that insects living in society, as the bee and the ant, maintain in their habitations a temperature higher than that of the open air, the fact had never yet been established that individual insects of every kind possess a more elevated temperature than that of the medium in which they reside, and that in each species the degree of elevation varies in the different stages of their existence. He was first led to study the temperature of insects in consequence of the curious results which he had met with in some observations he had himself made, in the autumn of the year 1832, on a species of wild bee in its natural haunts, with a view to ascertain, as had been suggested to him by Dr. Marshall Hall, the relation between the temperature of these insects during their hybernation, and the irritability of their muscular fibre: but the fact of the existence of a higher temperature in individual insects had been ascertained by himself prior to these observations; the results of which observations, together with other facts connected with the physiology of insects, he subsequently communicated to Dr. M. Hall.

Since the time when the author has been engaged in the prosecution of this enquiry, some observations on the same subject have been published by Dr. Berthold, of Göttingen, who expresses it as his opinion that insects ought not to be regarded as cold-blooded animals, but who does not appear to have detected the existence of a temperature higher than the surrounding medium in any individual insect.

The author gives a detailed account of his observations on the temperature of insects in their several states of larva, pupa, and imago, from which it appears that those which possess the highest temperature are always volant insects, and are chiefly diurnal species, residing almost constantly in the open air. He shows that the larva has a lower temperature than the imago, and that the energy of its respiration is also less, regard being had to the activity of the insect, and to the size of its body. In lepidopterous insects the average elevation of temperature above that of its surrounding medium, is in the larva from 0°.9 to 1°.5; while in the imago it is from 5° to 10°. Among the hymenoptera it is from 2° to 4° in the larva, and in the imago from 4° to 15° or even 20°; but in all cases the amount of this elevation is shown to depend on the degree of activity, and the quantity of air respired during a given period. The author then enquires into the influence of various circumstances, such as inactivity, sleep, hybernation, and inordinate excitement, on the temperature of insects; and shows that the evolution of heat gradually diminishes in a degree corresponding to the length of time during which the insect remains in a state of repose, but that it is immediately increased as soon as the insect is roused into action. He adverts also to the remote cause of hybernation, which he ascribes, in every state of the insect, to accumulations of adipose matter, or of nutrient fluid, which, being stored up in the system, induce a plethoric state, from which the animal is aroused when this store of materials has been exhausted. A variety of experiments are related, tending to prove that a large proportion of the heat evolved by an insect, when in a state of great activity, is dissipated into the surrounding medium, and that the quantity of heat so generated bears definite relations to the habits, the locality, and the energy of respiration in each respective species of insect. Volant insects, he finds, have the highest temperature; and of these the diurnal bear a higher temperature than the crepuscular; next to these must be placed the diurnal terrestrial, and last of all the nocturnal terrestrial species.

In the next division of this paper the author considers the temperature of those insects which live in societies; and in particular of the humble bee and the hive-bee. His observations are confirmatory of many of those of Huber relating to the incubating habits of the former of these species; and he has farther ascertained that during the act of incubation the bees possess a voluntary power of generating heat, whereby the temperature of their bodies is raised, apparently for the purpose of imparting warmth to the young in the cells; that this process is accompanied by accelerated respiration; and that the amount of heat evolved is proportional to the quantity of air respired. The law established by Dr. Edwards in the case of the young of mammiferous animals, namely, that they possess less power of generating heat, and that for a certain time they are unable to maintain their usual temperature, is shown by the author to be equally applicable to the early stage of insect life, and also to the perfect insect immediately after its development from the pupa.

The temperature of the hive-bee is next examined, and it is shown, contrary to the statements of Reaumur, Huber, and others, that bees do not maintain a very high temperature in their hives during winter, but that they are disposed, when not disturbed by any occasional vicissitudes of atmospheric temperature, to assume the state of hybernation; although, on the other hand, when the bees are much disturbed, the temperature of the hive may, even in the midst of winter, become greatly raised. The temperature of the hive is lowest in January, and gradually increases up to the period of swarming, in May or June, after which time it diminishes. A table is given exhibiting the results of successive observations on the influence of the diminution of heat and of light which attended the progress of the annular eclipse of the sun on the 15th of May, 1836, on the temperature of the hive.

It appears from the enquiries of the author that different parts of the hive do not preserve the same relative heat among one another at different periods, and also that the amount of free heat in the hive is often 10° or 15°, even in the months of July and August.

The remaining division of the paper is devoted to the consideration of the connexion existing between the development of heat and the functions of respiration, circulation, and digestion. The state of the pulse during all the different stages of the larva until its metamorphosis into the pupa is examined with great minuteness, and the results are given in a tabular form. The author traces the rate of pulsation during different conditions of repose and activity, and the corresponding frequency of respirations, and finds that although there is a general accordance between the activity of these two functions, yet that the activity of respiration and the quantity of heat evolved do not depend primarily on the velocity of the circulation, but that under all circumstances the quantity of heat developed is exactly proportional to the quantity of respiration. While the insect is feeding, and digestion is going on, the evolution of heat increases, and while it is fasting it diminishes; but this diminution has a limit, whereas increased respiration is invariably attended by increased heat. Gaseous matter is exhaled in great abundance from the surface of the body of an insect, and contributes to regulate and equalize its temperature; but the quantity diminishes in proportion to the length of time during which it has been deprived of food. The author maintains that animal heat is not an effect of mere nervous influence, either general or ganglionic; an opinion which he derives from the following considerations: first, that in many insects in which considerable degrees of heat are evolved, and the respiration is energetic, the nervous system is small compared with that of others in which the respiration is less vigorous; and secondly, that if the evolution of animal heat were dependent on the existence of ganglia, the leech ought to generate more heat than the larva of the lepidoptera, since it has a much greater number of ganglia. Hence he is disposed to draw the general conclusion that animal heat results directly from the changes which take place during respiration; and that the reason why so large a quantity passes off so rapidly from the body of an insect is because it does not become latent, since the circulating fluid, unlike what takes place in the higher animals, is neither completely venous nor completely arterial, but of a character intermediate between both.

Twenty-one tables are annexed exhibiting the records of the experiments referred to in the paper on the respiration, temperature, and circulation of insects.

*Proceedings of the Royal Society*, No. xxix.

---

*On the Brain of the Negro, compared with that of the European and the Ourang-Outang.* By FREDERICK TIEDEMANN, M.D., Professor of Anatomy and Physiology in the University of Heidelberg. (*Read before the Royal Society, June* 9, 1836.)

IT has long been the prevailing opinion among naturalists that the Negro race is inferior, both in organization and in intellectual powers, to the European; and that, in all the points of difference, it exhibits an approach to the Monkey tribes. The object of the present paper is to institute a rigid enquiry into the validity of this opinion. The author has, for this purpose, examined an immense number of brains of persons of different sexes, of various ages, and belonging to different varieties of the human race, both by ascertaining their exact weight, and also by accurate measurement of the capacity of the cavity of the cranium; and has arrived at the following conclusions. The weight of the brain of an adult male European varies from 3lbs. 3 oz. to 4lbs. 11 oz. troy weight: that of the female weighs, on an average, from four to eight ounces less than that of the male. The brain usually attains its full dimensions at the age of seven or eight; and decreases in size in old age. At the time of birth, the brain bears a larger proportion to the size of the body than at any subsequent period of life, being then as one-sixth of the total weight; at two years of age it is one-fourteenth; at three, one-eighteenth; at fifteen, one twenty-fourth; and in the adult period, that is, from the age of twenty to that of seventy, it is generally within the limits of one thirty-fifth and one forty-fifth. In the case of adults, however, this proportion is much regulated by the condition of the body as to corpulence; being in thin persons from one twenty-second to one twenty-seventh, and in fat persons often only one fiftieth, or even one hundredth of the total weight of the body. The brain has been found to be particularly large in some individuals possessed of extraordinary mental capacity. No perceptible difference exists either in the average weight or the average size of the brain of the Negro and of the European: and the nerves are not larger, relatively to the size of the brain, in the former than in the latter. In the external form of the brain of the Negro a very slight difference only can be traced from that of the European; but there is absolutely no difference whatsoever in its internal structure, nor does the Negro brain exhibit any greater resemblance to that of the ourang-outang than the brain of the European, excepting, perhaps, in the more symmetrical disposition of its convolutions.

Many of the results which the author has thus deduced from his researches are at variance with the received opinions relative to the presumed inferiority of the Negro structure, both in the conformation and relative dimensions of the brain; and he ascribes the erroneous notions which have been hitherto entertained on these subjects chiefly to prejudice created by the circumstance that the facial angle in the negro is smaller than in the European, and consequently makes, in this respect, an approach to that of the ape, in which it is still farther diminished. The author denies that there is any innate difference in the intellectual faculties of these two varieties of the human race; and maintains that the apparent inferiority of the Negro is altogether the result of the demoralizing influence of slavery, and of the long-continued oppression and cruelty which have been exercised towards this unhappy portion of mankind by their more early civilized, and consequently more successful competitors for the dominion of the world.

*Proceedings of the Royal Society*, No. xxvi.

---

### *On the Voluntary and Instinctive Actions of Living Beings.*
By William B. Carpenter, Esq. m.r.c.s. &c.

This paper, the substance of which was originally read to the Royal Medical Society of Edinburgh, of which society Mr. Carpenter was the senior president, is of great interest both in a physiological and psychological point of view, and proves the author's powers in the investigation of such subjects, to be of a very superior order. We expect yet greater things from his acute, well-disciplined and well-directed mind. We can only, in this place, find room for the general summary of the positions advanced in the memoir, to which we refer the reader.

"1. Many living tissues possess the property of contractility upon the application of a stimulus.

"2. This contractility is especially manifested by the irritable parts of certain vegetables; and results in all these cases from the action of a stimulus either *directly* applied or conveyed through the circulating system.

"3. This contractility is also especially manifested by the muscular fibre of animals, and may in them be called into play not only by the stimuli which act upon plants, but by another of a peculiar nature, commonly denominated nervous influence.

"4. Whatever actions (whether consisting of visible motions or not) are performed by the tissues of plants, may be regarded as the direct respondence of their organism to external stimuli, and as solely connected with their organic life.

"5. All the actions (whether consisting of visible motions or not) essential to the organic life of animals, are in like manner produced by the *immediate* action of external stimuli;* and being entirely involuntary, may be called *organic instincts*. Under this head are included (besides many less apparent changes,) the motions of the heart and alimentary canal.

"6. The first office of the nervous system is to convey to a distant part the *impressions* made upon it, and to produce (by its *stimulation* of the contractile tissues) motions necessarily connected with them. These actions, being purely instinctive and involuntary, may be called *excito-motor instincts*.

"7. All that is required for these manifestations is the completeness of the circle of concentric and excentric nerves. In vertebrated animals, the cerebro-spinal axis and nerves proceeding from it are of course solely concerned in this function.

"8. In the lowest animals possessing a simple nervous system, these actions make up the greatest part of the sum of the life of the individual; and in the higher, they are immediately connected with the supply of the organic instincts.

"9. Where a more complicated nervous system exists, the impressions give rise to mental changes termed *sensations*, the seat of which is some part of the cerebral mass in the vertebrata, and probably the ganglia connected with the nerves of sense in the invertebrata. With various sensations, certain involuntary motions are instinctively associated; but as *sensation*, a mental change in the sensorium, cannot immediately give rise to *stimulation*, an organic change at the extremities of the nerves, a *motive action* must be propagated from the sensorium along the nervous conductors, and this cannot result from an external impression wherever sensation does not exist. The instinctive actions thus resulting are still purely involuntary, although they may be controlled in man by the higher power of the will. Certain habitual actions may come to take place nearly in the same circle, which may be called that of *sensori-motor instincts*.

"10. Voluntary actions require *perceptions* in addition to sensations and impressions. Perceptions give rise to mental processes terminating in *volition*, which produces motive action and stimulation. Volition is of course confined to the brain, and probably to the cerebral lobes.

"11. No distinct division of the spinal marrow is necessary for the performance of the excito-motor, or sensori-motor instinctive actions. The same nervous matter may act as a conductor either to the influence of the will (motive action) or to that of a simple impression (stimulation.)

"12. As all the nerves of sensation terminate ultimately in the cerebro-spinal axis, (the spinal cord and its prolongations as far as the *crura cerebri* and *corpora quadrigemina*) and all the nerves of motion arise from it, it follows that all motions must be the result of some stimulus applied to this system; and this stimulus may either be given by an impression from without, or by a mental change within (or in the brain above.) If we regard the cineritious matter of the brain as a seat of consciousness, sensation, the intellectual powers, and volition, the medullary portion of the hemispheric ganglia being merely a conductor, (a position which I do not wish to be understood as advocating,) it necessarily follows, from the positions previously taken, that the sensory and motor tracts in the brain simply convey *upwards* to the cineritious matter, the influence of the impression existing in the cerebro-spinal axis, and *downwards* the motive action resulting either immediately from sensation, or from volition." *Edinburgh Journal. July*, 1837.

* The term *external* is here employed in the usual metaphysical sense, implying something distinct from *mental* action. The stimulus may originate in the corporeal organism itself.

*On Unity of Function in Organized Beings.* By W. B. Carpenter, Esq.

This is another paper by the same author, and possesses the same ingenious and philosophical character. As it has less relation to human physiology, we must content ourselves with a mere reference to it: but its perusal will gratify all those who have been accustomed to regard physiology generally, as it relates to animated beings. The chief object of the author in this paper is to apply to *function* one of the laws propounded by Von Bär with regard to structure, namely, that, "a special function arises only out of one more general, and this by a gradual change;" to which Mr. Carpenter adds a second, namely, that "in all cases where the different functions are highly specialized, the general structure retains, more or less, the primitive community of function which originally characterized it." "The time has long gone by (says Mr. C.) when similarity in function and external form were considered sufficient for the recognition of analogies between organs; anatomists are now aware of the necessity of resting their comparison upon the elementary structure of organs, their connexions with each other, and the changes they undergo during the progress of their development. Neither of these grounds of judgment can be safely trusted to alone; whilst, combined with each other, they furnish a body of evidence which is quite irresistible."

*Edinburgh Philosophical Journal. July,* 1837.

